# Supplementary material for: Movement in low gravity environments (MoLo) programme–The MoLo-L.O.O.P. study protocol
Source: PLoS One. 2022 Nov 23;17(11):e0278051. doi: 10.1371/journal.pone.0278051 (PMC9683620; doi:10.1371/journal.pone.0278051)
Supplement: Attachment — Submitted filename: Response to Reviewers.docx [file pone.0278051.s002.docx]

**Response to Reviewers**

*Editor Comments:*

1. Please ensure that your manuscript meets PLOS ONE's style requirements, including those for file naming. The PLOS ONE style templates can be found at

<https://journals.plos.org/plosone/s/file?id=wjVg/PLOSOne_formatting_sample_main_body.pdf> and

[https://journals.plos.org/plosone/s/file?id=ba62/PLOSOne_formatting_sample_title_authors_affiliations.pdf](https://eur05.safelinks.protection.outlook.com/?url=https%3A%2F%2Fjournals.plos.org%2Fplosone%2Fs%2Ffile%3Fid%3Dba62%2FPLOSOne_formatting_sample_title_authors_affiliations.pdf&data=05%7C01%7Cnolan.herssens%40esa.int%7Ca5a5d37661f647ebd9cd08dab5ce27c6%7C9a5cacd02bef4dd7ac5c7ebe1f54f495%7C0%7C0%7C638022194284916222%7CUnknown%7CTWFpbGZsb3d8eyJWIjoiMC4wLjAwMDAiLCJQIjoiV2luMzIiLCJBTiI6Ik1haWwiLCJXVCI6Mn0%3D%7C3000%7C%7C%7C&sdata=7u8o05Nxp4%2BUIy2TAiz1YOuh2XX%2BZHe4KWKCBk0YFI4%3D&reserved=0)

🡪 We have checked and made some changes to the style of the revised manuscript in order to adhere to the PLOS ONE style requirements. Please note that changes to the formatting have not been highlighted in the revised manuscript.

2. We note that the grant information you provided in the ‘Funding Information’ and ‘Financial Disclosure’ sections do not match.

When you resubmit, please ensure that you provide the correct grant numbers for the awards you received for your study in the ‘Funding Information’ section.

🡪 We have checked both the “Funding information” and “Financial Disclosure” sections and both sections should now contain the same information.

*This work was supported by the European Space Agency: ESA contract No. 4000123348/18/NL/MH with the University of Bath; ESA contract No. 4000133724/21/NL/AT with the University of Applied Sciences Aachen.*

*The "Locomotion On Other Planets (L.O.O.P.): Hypogravity Analogue" of the University of Milan is supported by the European Space Agency as an ESA ground-based facility through the Continuously Open Research Announcement Opportunity for Ground-Based Facilities (ESA-CORA-GBF), ESA contract No. 4000137794/22/NL/PA/pt.*

*This study was funded by CAMERA, the RCUK Centre of the Analysis of Motion, Entertainment Research and Applications, EP/M023281/1.*

*Authors DG and TW are employed by KBR GmbH on behalf of the European Space Agency. The funder KBR GmbH provided support in the form of salaries for the authors DG and TW but did not have any role in the study design, data collection, and analysis, decision to publish, or preparation of the manuscript.*

*The funders had and will not have a role in study design, data collection and analysis, decision to publish, or preparation of the manuscript.*

3. We note that you have indicated that data from this study are available upon request. PLOS only allows data to be available upon request if there are legal or ethical restrictions on sharing data publicly. For more information on unacceptable data access restrictions, please see <http://journals.plos.org/plosone/s/data-availability#loc-unacceptable-data-access-restrictions>.

In your revised cover letter, please address the following prompts:

a) If there are ethical or legal restrictions on sharing a de-identified data set, please explain them in detail (e.g., data contain potentially sensitive information, data are owned by a third-party organization, etc.) and who has imposed them (e.g., an ethics committee). Please also provide contact information for a data access committee, ethics committee, or other institutional body to which data requests may be sent.

b) If there are no restrictions, please upload the minimal anonymized data set necessary to replicate your study findings as either Supporting Information files or to a stable, public repository and provide us with the relevant URLs, DOIs, or accession numbers. For a list of acceptable repositories, please see <http://journals.plos.org/plosone/s/data-availability#loc-recommended-repositories>.

We will update your Data Availability statement on your behalf to reflect the information you provide.

🡪 We have updated the Data Availability statement in the online submission environment to “Yes – any pilot data reported in this submission are fully available.” as the pilot data used to perform the sample size calculations are available as Supporting Information (S1 File).

We have also addressed this within the revised cover letter as requested.

4. Please review your reference list to ensure that it is complete and correct. If you have cited papers that have been retracted, please include the rationale for doing so in the manuscript text, or remove these references and replace them with relevant current references. Any changes to the reference list should be mentioned in the rebuttal letter that accompanies your revised manuscript. If you need to cite a retracted article, indicate the article’s retracted status in the References list and also include a citation and full reference for the retraction notice.

🡪 We have reviewed the reference list and have made the following changes:

- We have added the following reference as suggested by Reviewer #1:

[40]: McDonnell J, Zwetsloot KA, Houmard J, DeVita P. Skipping has lower knee joint contact forces and higher metabolic cost compared to running. Gait Posture 2019;70:414–9. <https://doi.org/10.1016/j.gaitpost.2019.03.028>.

- We have removed the following references as the manuscript is still under preparation and is not essential to the figure it is pointing to, as the figure has been made specifically for the protocol submission, and is not included in the paper referred to:

*Previously: [64]	Cowburn J, Serrancolí G, Pavei G, Minetti A, Salo A, Colyer S, et al. The Biomechanical Handbook: a novel computational framework for the estimation of internal musculoskeletal loading in hypogravity. Manuscr Prep 2021.*

*Reviewer #1:*

Reviewer #1: The paper describes in details a research protocol to study locomotion and movements in low gravity environments. The manuscript is well written and the topic of great interests. The renewed interest in space exploration is providing new momentum to many research topics, including physio mechanics of locomotion.

The introduction is correctly focused on the topics of the investigation and the state of the art. References are sound. As one of the objective would be the estimation of joint reaction forces, maybe it would be worth to take into account the results of McDonnell et al. (2019, Gait & Posture 70, 414-419).

🡪 Thank you for this comment, we have read the paper the reviewer has referred to and have added a statement discussing the relevant information within the introduction, lines 121-130.

The protocol is very well designed. I only have one remark, or one question, about the range of speed and gaits. I noticed that the skipping trials would start at 1.94 m/s, and I'm wondering about the reasons of this choice. At lunar gravity the walk-run transition is estimated at a speed around 1 m/s, and it is known, from previous experiments and from the Apollo missions reports, that when astronauts can't walk, they start skip.
It is also true that the walk-skip gait change would imply an abrupt increase of the speed. However, I would have expected a wider range of speed for skipping trials.

🡪 Thank you for this comment, we agree with the reviewer that at lunar gravity skipping will be adopted as the gait of choice at lower speeds than the two here analysed. We were forced to address skipping with just those two speeds due to a time restriction (the protocol would be longer of about twenty minutes) and also the knowledge that at 1, 0.7 and 0.5 g the skipping gait at 1.39 m/s is not that natural and meaningful. However, we will do our best to add the 1.39 m/s acquisition to Moon and Mars gravity, according to this right suggestion and in order to collect more interesting data.
